# Regulation of transforming growth factor is involved in the efficacy of combined 5-fluorouracil and interferon alpha-2b therapy of advanced hepatocellular carcinoma

**DOI:** 10.1038/s41420-018-0040-y

**Published:** 2018-03-12

**Authors:** Youhei Okada, Ting Wang, Kazuhiro Kasai, Kazuyuki Suzuki, Yasuhiro Takikawa

**Affiliations:** Division of Gastroenterology and Hepatology, Department of Internal Medicine, Iwate Medical University, Morioka, Iwate Japan

## Abstract

Transforming growth factor-beta (TGF-β) is critical in cancer cell invasion and metastasis. The effects of a treatment that targets TGF-β using the combination of interferon alpha (IFNα)-2b and 5-fluorouracil (5-FU) are unknown. Here, we show that the serum levels of TGF-β1 prior to the therapy correlate with increased maximum tumor diameter, which is significantly (*p* < 0.01) decreased after the combination therapy. 5-FU increased both the expression and secretion levels of TGF-β1 in hepatoma cells, but not in normal hepatocytes. The combination of 5-FU and IFNα-2b synergistically affected cell death. However, a TGF-β1 specific inhibitor did not affect the anti-tumor activity of 5-FU. 5-FU inhibited the phosphorylation of SMAD2 and reduced the total protein levels of SMAD2, SMAD4, and pINK4b. Conversely, 5-FU stimulated the phosphorylation of extracellular signal-regulated kinase (ERK)1/2. Accordingly, the protein levels of E-cadherin and claudin-1 were reduced in 5-FU-treated cells. The combination of 5-FU and IFNα-2b, and the inhibition of ERK1/2 by a specific inhibitor neutralized the effects of 5-FU on TGF-β-related signaling molecules and restored their protein levels to those observed in the control. Interestingly, the phosphorylated protein levels of SMAD2 and the total protein levels of E-cadherin and p15INK4b were increased in 5-FU-stimulated HuH-7 cells, but not in Hep G2 cells. Our data suggest that the higher efficacy of the 5-FU and IFNα-2b combination therapy was associated with the regulation of TGF-β expression, secretion, and the signals mediated by it.

## Introduction

Common hepatocellular carcinoma (HCC) can be treated using diverse approaches that include hepatectomy, transcatheter arterial chemo-embolization (TACE), and radiofrequency ablation (RFA). However, there is no effective therapy for advanced HCC with portal vein tumor thrombosis (PVTT)^1^. A combination therapy of interferon alpha (IFNα)-2b and 5-fluorouracil (5-FU), which was developed in the early Twenty-first century^2, 3^, is being reconsidered as a treatment of advanced HCC. We recently reported the improved survival rate of patients with advanced HCC featuring portal venous invasion using this combination therapy^4, 5^. However, the cumulative survival rate was < 50% at 36 months, which suggested that the treatment is not suitable or produces a poor response for some patients. Better understanding of the mechanistic details of this therapy would help in developing highly effective treatments for advanced HCC and in predicting the causes of adaptation to the therapy.

Several reports have attempted to explain the mechanism of this combination chemotherapy. IFN receptors may be involved in inducing the apoptosis of cancer cells that has been observed with this combination chemotherapy^6, 7^. Another possibility is the inhibition of the immunosuppression of cancer cells via tumor necrosis factor-related apoptosis-induced ligand (TRAIL) signaling^8, 9^. Angiopoietin 2-related anti-angiogenesis might also have a role in the success observed with this treatment^10, 11^. However, these studies focused on the individual characteristics that appeared during the progression of advanced HCC, and did not determine the factors affecting the general efficacy of the combination therapy, such as targeting of key molecules or the associated signaling pathways.

As a member of the transforming growth factor-beta superfamily, TGF-β activates the SMAD-dependent signaling pathway, which induces apoptosis and inhibits the proliferation of epithelial cells. Therefore, TGF-β has long been considered an anti-oncogenic molecule^12^. However, recent research has revealed that mutations in certain components of the TGF-β signaling pathway exist in many cancers, especially in TGF-β receptor 2 and SMADs^13–15^, which disrupt signal transduction and the subsequent inhibition of proliferation and apoptosis. In these mutants, TGF-β activates members of the mitogen-activated protein kinase (MAPK) pathway, such as extracellular signal-regulated kinase 1 and 2 (Erk1/2, or p44/42), c-Jun N-terminal kinases (JNKs), and p38 isoforms (p38 MAPK). This TGF-β-induced SMAD-independent mitogenic signaling protects cancer cells from apoptotic cell death and induces epithelial–mesenchymal transition (EMT), which renders the tumor cells metastatic^13, 16, 17^. In addition, TGF-β is secreted excessively by several cancerous tissues, and contributes to the maintenance of the cancer stem cell population, immunosuppression, and angiogenesis^18, 19^.

Considering the importance of TGF-β in cancer development, we evaluated the relationship between the serum levels of TGF-β and the efficacy of the IFNα-2b/5-FU combination therapy. We also studied the effects of 5-FU with or without IFNα-2b on the regulation of TGFβ.

## Results

### Serum levels of TGF-β1 in patients with advanced HCC

**Fig. 1:**
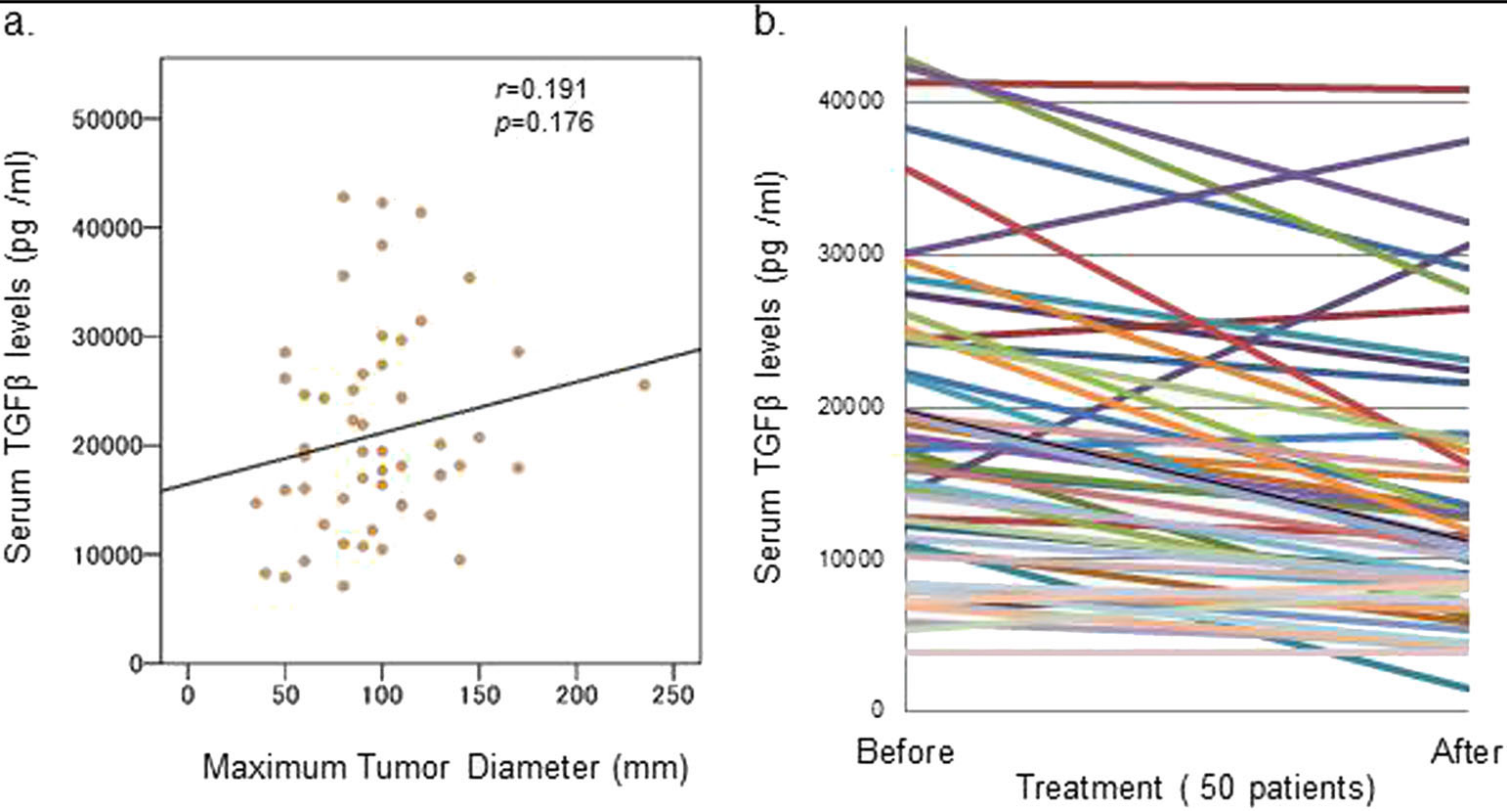
Detection of TGF-β1 serum levels in patients with advanced HCC. **a** TGF-β1 levels before the treatment were used to analyze the correlation with the maximum tumor diameter. A *p*-value and its correlation were calculated in Microsoft Excel 2010. **b** TGF-β1 serum levels in patients before and after treatment were determined according to the manufacturer’s instructions. Fifty patients were analyzed. Statistical comparisons were performed using a paired *t*-test. A *p*-value < 0.05 was considered to be significant

On the basis of our previous results that the mean survival rate of patients is improved after the IFNα-2b/5-FU combination therapy^4, 5^, we first quantified the serum levels of TGF-β1 in patients with advanced HCC before and after the combination therapy to determine whether the efficacy of the therapy correlated with the regulation of TGF-β levels. Serum levels of TGF-β1 before the therapy showed a tendency towards correlation with increased maximum tumor diameter, which is used to evaluate the progress of HCC (Fig. 1a). Therapy was associated with significantly decreased serum levels of TGF-β1 (*p* < 0.01; Fig. 1b). Seventeen patients had a background of hepatitis C virus (HCV) infection. Ten of the 17 patients improved and seven worsened, as determined by a computed tomography (CT) scan 1 month after receiving the combination therapy. In these 17 patients, the serum levels of TGF-β1 decreased following the therapy, with no significant difference in the therapy-related change in TGF-β1 levels between the patients who improved and worsened (Supplemental Data).

### Effects of 5-FU or IFNα-2b alone and in combination on TGF-β expression in Hep G2 cells

**Fig. 2:**
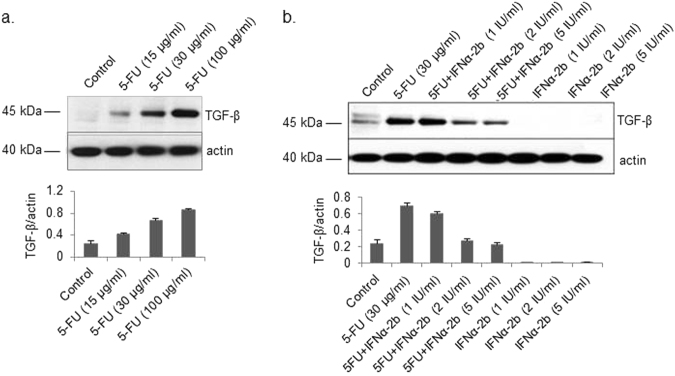
Effects of 5-FU and IFNα-2b alone and in combination on TGF-β levels in Hep G2 cells. Cells were treated with 0, 15, 30, and 100 µg/mL 5-FU for 24 h (**a**) or with 30 µg/mL 5-FU, IFNα-2b (1, 2, 5 IU/mL), or 5-FU and IFNα-2b for 24 h (**b**). TGFβ levels in the cell lysates were determined by western blotting. Results are expressed as the means ± SD (*n* = 3)

The foregoing data prompted an in vitro assessment of whether the efficacy of the combination therapy results from a synergistic effect of 5-FU and IFNα-2b on the regulation of TGF-β. Unexpectedly, we found that 5-FU treatment stimulated and increased the protein levels of TGF-β in the lysates of cultured Hep G2 cells in a dose-dependent manner (Fig. 2a). In contrast, no TGF-β was detected in cells treated with IFNα-2b (Fig. 2b). The combination of 5-FU and 1–5 IU/mL IFNα-2b completely inhibited the expression of TGF-β induced by 5-FU (Fig. 2b).

### Effects of 5-FU treatment alone, or in combination with IFNα-2b or TGF-β receptor inhibitor, on the viability and apoptosis of Hep G2 cells

**Fig. 3:**
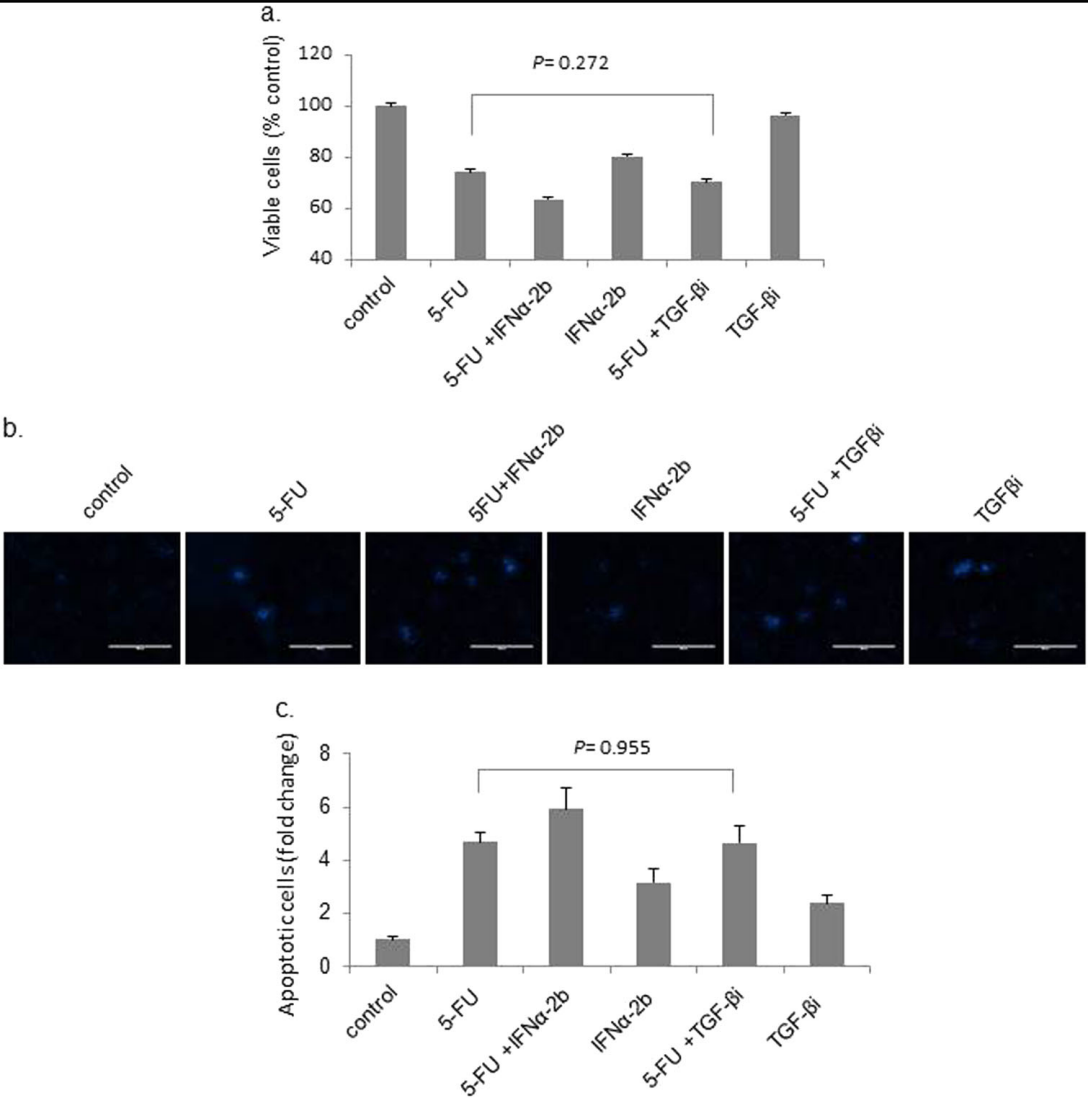
Effects of 5-FU treatment alone, or in combination with IFNα-2b or TGF-β receptor inhibitor, on the viability and apoptosis of Hep G2 cells. **a** Cells were treated with 15 µg/mL 5-FU, 2 IU/mL IFNα-2b, or a combination of both for 48 h. In additional experiments, cells were pretreated with 10 µM SB-431542 (a TGF-β inhibitor) before treatment with 5-FU. The number of viable cells was evaluated by checking the optical density at 450 nm. **b** Cells were given the same treatment as described in **a**; thereafter, the apoptotic cells were stained, photographed (scale = 400 μM), and counted (**c**) as described in the materials and methods section. The statistical analysis was carried out using one-way ANOVA-POST HOC (Tukey’s HSD) analysis. A *p*-value < 0.05 was considered to be significant. Results are expressed as the means ± SD (*n* = 3)

Since 5-FU is a potent anti-tumor reagent, we quantified cell viability to determine whether the increased levels of TGF-β stimulated by the 5-FU treatment contributed to the anti-tumor activity of 5-FU. Treatment with 5-FU significantly decreased the number of viable cells (Fig. 3a) and induced cell apoptosis (Fig. 3b, c), and the combination of 5-FU and IFNα-2b generated a synergistic effect, consistent with previous reports (Fig. 3a-c)^20–22^. However, treatment of the cells with SB-431542, a potent and specific inhibitor of the TGF-β1 receptor, prior to 5-FU treatment did not significantly change the effects of 5-FU treatment alone (*p* > 0.05; Fig. 3a–c).

### Effects of 5-FU on TGF-β-related signaling molecules

**Fig. 4:**
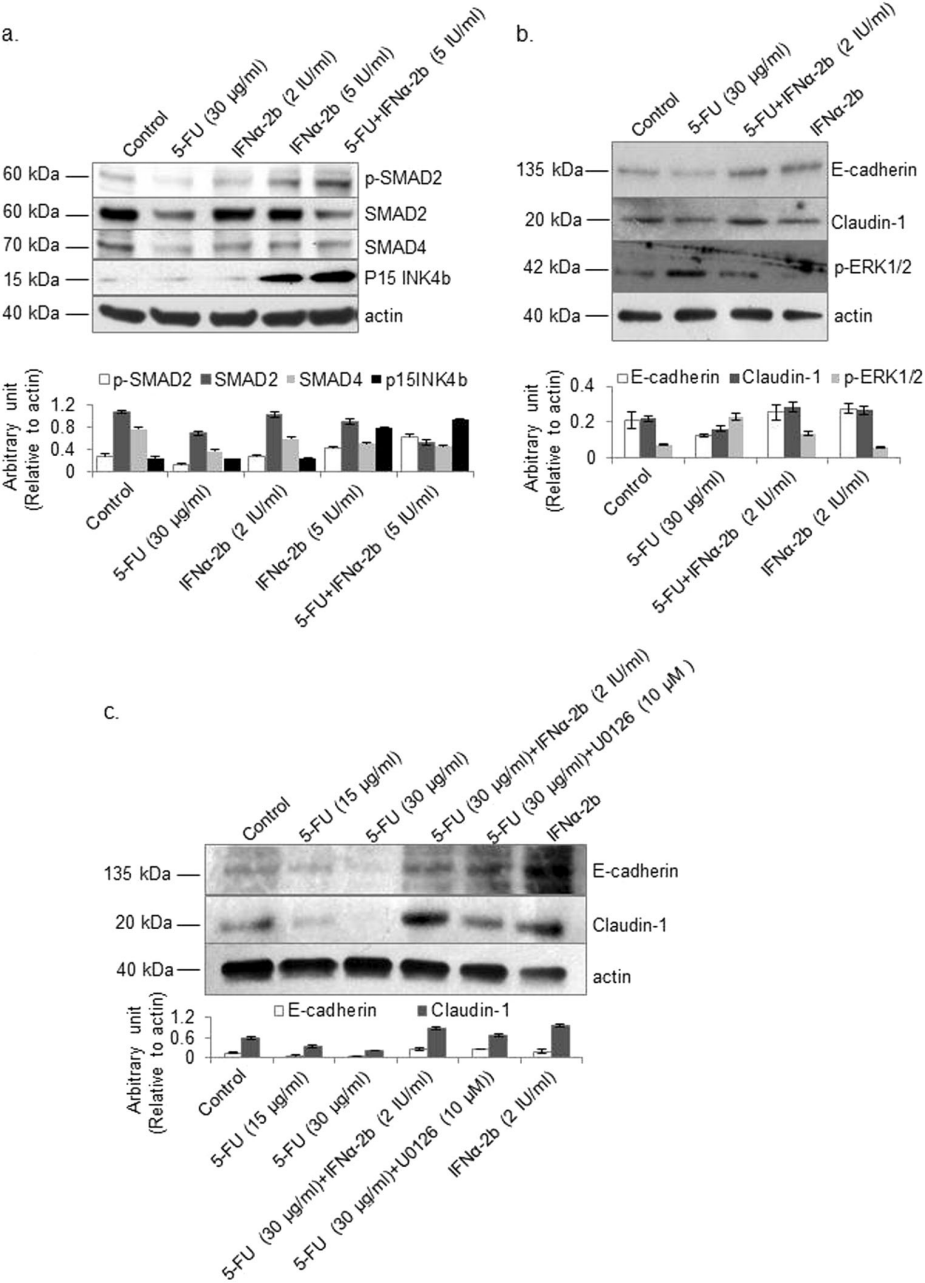
Effects of 5-FU and IFNα-2b alone and in combination on TGF-β-mediated signaling. Hep G2 cells were treated with 5-FU and/or IFNα-2b at the indicated concentrations for 24 h. **a** The protein levels of p-SMAD2, SMAD2, SMAD4, and p15INK4b were detected by western blotting. **b** In another experiment, levels of EMT molecules including E-cadherin, claudin-1, and p-ERK1/2 were detected by western blotting as described in the materials and methods section. **c** Besides the treatment of 5-FU and IFNα-2b alone and in combination, the ERK1/2 inhibitor U0126 was used to clarify the role of ERK1/2 in the regulation of E-cadherin and claudin-1 by 5-FU. Actin was detected as an internal control. The quantitative data are presented as the means ± SD (*n* = 3)

The effect of 5-FU on TGF-β-induced apoptosis signals was investigated. 5-FU inhibited the phosphorylation of SMAD2 and reduced the total protein levels of pINK4b (Fig. 4a). Conversely, 5-FU stimulated the phosphorylation of ERK1/2, a TGF-β-induced EMT molecule. During the progression of EMT, tight junction proteins, like claudins and adherens junction proteins like E-Cadherin are usually downregulated^23, 24^. Consistent with these observations, we observed that the protein levels of E-cadherin and claudin-1 were reduced in the cells treated with 5-FU (Fig. 4b). On the other hand, IFNα-2b stimulated the phosphorylation of SMAD2, but not ERK1/2, increased the total protein levels of pINK4b, and did not affect the levels of E-cadherin and claudin-1. The combination of 5-FU and IFNα-2b inhibited the effects of 5-FU on the aforementioned TGF-β-related signaling molecules (Fig. 4a, b). In addition, similar to the effects of IFNα-2b, U0126 (a MEK-specific inhibitor and an upstream effector of ERK1/2) inhibited the negative effect of 5-FU on the regulation of E-cadherin and claudin-1 (Fig. 4c). The other EMT molecules, vimentin and slug showed no expression. In addition, there was no obvious change in the expression of snail, TCF8/ZEB1, ZO-1 and β-catenin upon treatment with 5-FU in comparison to controls (data not shown).

### 5-FU increases expression and secretion levels of TGF-β1 in Hep G2 and HuH-7 cells

**Fig. 5:**
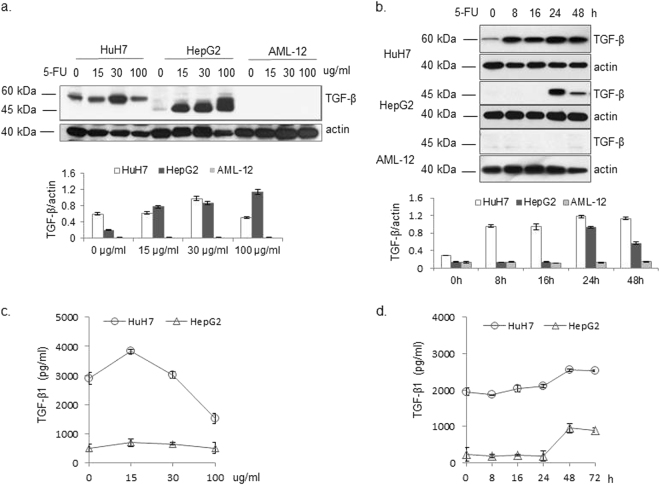
Cell-dependent regulation of TGF-β expression and secretion by 5-FU. HuH-7, Hep G2, and AML-12 cells were treated with different concentrations of 5-FU as indicated (**a**), and 30 µg/mL 5-FU for the indicated time periods (**b**). TGF-β was detected by western blotting. TGF-β secretion was measured in HuH-7 and Hep G2 cells as described in Materials and Methods (**c**, **d**). Results are expressed as the means ± SD (*n* = 3)

**Fig. 6:**
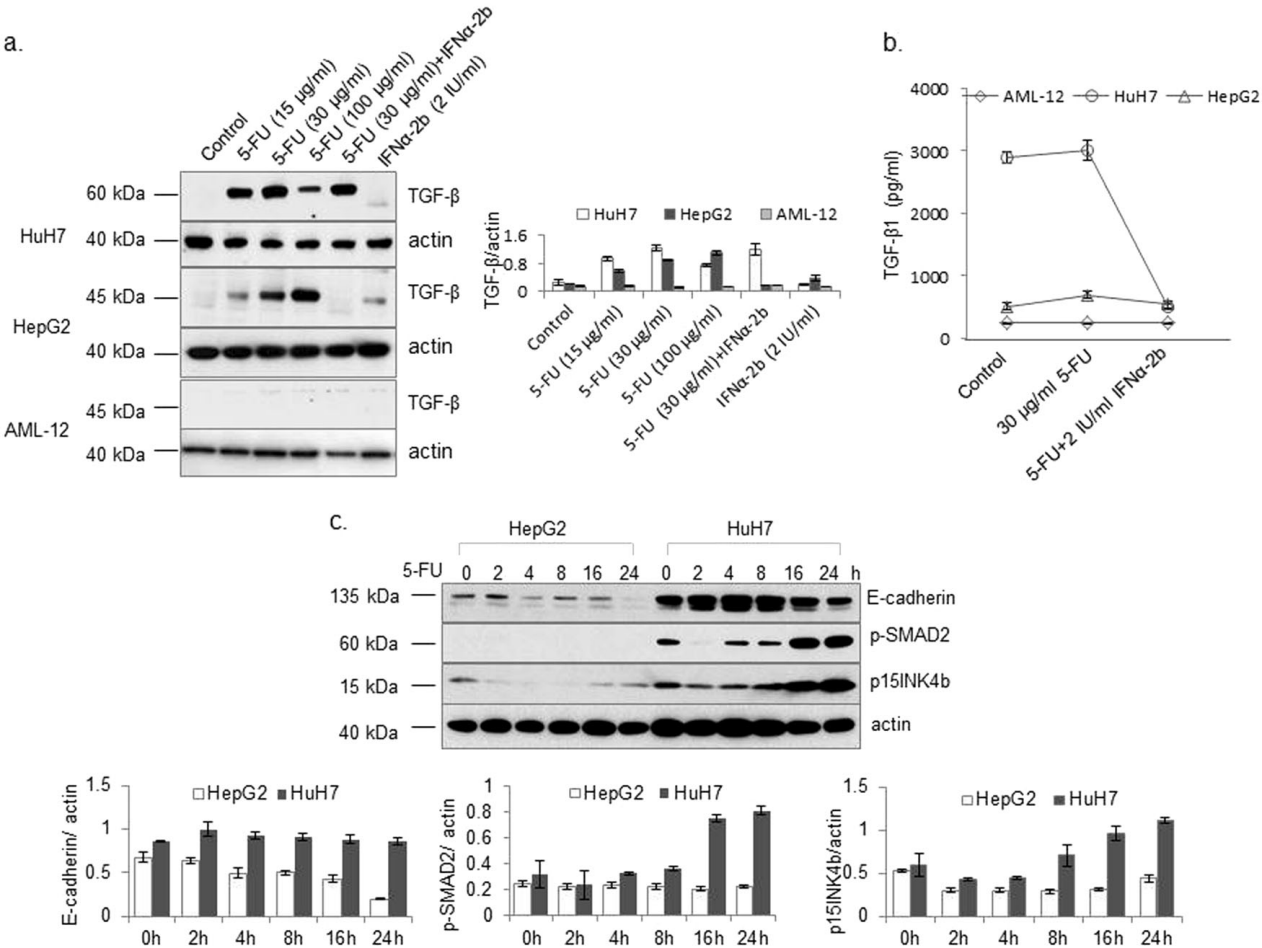
Responses of different cells to 5-FU, IFNα-2b, and combined treatment with respect to TGF-β levels and TGF-β-mediated signaling. Hep G2, HuH-7, and AML-12 cells were individually treated with 5-FU, IFNα-2b, and a combination of 5-FU and IFNα-2b for 24 h. TGF-β levels in cell lysates (**a**) and culture supernatants (**b**) were detected. (**c**) Both Hep G2 and HuH-7 cells were treated with 5-FU for different periods as indicated. The levels of E-cadherin, p-SMAD2, and p15INK4b were detected by western blot. Actin was detected as an internal control. Results are expressed as the means ± SD (*n* = 3)

We also compared the effects of 5-FU on the expression and secretion of TGF-β in Hep G2 and HuH-7 hepatocarcinoma cells, and normal alpha mouse liver AML -12 hepatocytes. Time- and dose-course experiments revealed increased TGF-β levels in 5-FU treated Hep G2 and HuH-7 cells. In HuH-7 cells, the levels of TGF-β levels peaked upon treatment with 30 µg/mL 5-FU (Fig. 5a), whereas there was a progressive increase in TGF-β levels in Hep G2 cells. Conversely, a TGF-β peak appeared in Hep G2 cells that were treated for 24 h with 5-FU, whereas there was a progressive increase in TGF-β levels for up to 48 h in HuH-7 cells (Fig. 5b). We further compared the TGF-β secretion pattern of these hepatoma cell lines. The baseline level of TGF-β in the supernatant fraction of cultured HuH-7 cells was higher than that of Hep G2 cells. The peak in TGF-β secretion occurred upon a 48-h treatment of either cell line with 15 µg/mL 5-FU (Fig. 5c, d). In contrast, there was no expression (Fig. 5a, b) or secretion (Fig. 6b) of TGF-β in normal AML-12 hepatocytes with or without 5-FU treatment.

### Differences in the effects of 5-FU and IFNα-2b combination treatment on TGF-β regulation between Hep G2 and HuH-7 cells

The combination of 5-FU and IFNα-2b decreased both the expression and secretion of TGF-β in Hep G2 cells compared to cells treated with 5-FU alone. However, in HuH-7 cells, the combination therapy decreased only the secretion of TGF-β, with no effect on the protein levels (Fig. 6a, b). Interestingly, the phosphorylated protein levels of SMAD2 and the total protein levels of E-cadherin and p15INK4b were increased in 5-FU-stimulated HuH-7 cells, in contrast to the inhibition of these TGF-β-induced signaling molecules in 5-FU-stimulated Hep G2 cells (Fig. 6c).

## Discussion

Elevation in the serum levels of TGF-β or TGF-β expression have been reported in various cancers, and particularly during the progression of advanced HCC^25–30^. TGF-β1 is a useful biomarker of prostate cancer and small HCC^28, 29^. Presently, although no significant difference was found, possibly because all the subjects were stage IV, serum TGF-β levels tended to correlate positively with maximum tumor diameter. More importantly, TGF-β levels decreased in patients who were administered a combined chemotherapy of 5-FU and IFNα-2b, accompanied by a high efficacy of the therapy with a mean survival rate of 29.9 months^4^. The data suggest that the reduction in TGF-β levels may be useful to predict the efficacy of the combination therapy.

Surprisingly, 5-FU increased both the protein and secretion levels of TGF-β, and regulated the TGF-β-induced signaling in Hep G2 and HuH-7 hepatoma cells, but not in normal hepatocytes. A recent study reported that 5-FU can activate the TGF-β pathway and reasonably upregulate the expression of TGF-β in an autocrine manner in drug-resistant colorectal carcinoma cells^31^. The association between 5-FU and TGF-β signaling has also been described in human HCC cells^32^. It is possible that 5-FU regulates TGF-β signaling indirectly via poly (ADP-ribose) polymerase-1 (PARP-1), a nuclear enzyme that is conventionally linked to DNA repair^33, 34^. How 5-FU regulates TGF-β and its pathway in HCC cells, and whether its function is limited only to cancer cells is unclear and requires further investigations.

TGF-β regulates both apoptosis and EMT of hepatocytes. However, resistance of hepatoma cells to TGF-β-induced apoptosis has been reported^35^. In the present study, a specific TGF-β receptor inhibitor did not antagonize the effects of 5-FU on Hep G2 cell viability and apoptosis. Additionally, although the combination of 5-FU and IFNα-2b was more lethal to cancer cells than 5-FU alone, TGF-β expression and secretion decreased in cells treated with the drug combination compared to cells treated with 5-FU alone. Our data indicate that the increase in TGF-β levels does not contribute to the anti-cancer activity of 5-FU in Hep G2 cells. Furthermore, we found that 5-FU inhibited TGF-β-mediated apoptosis signaling but stimulated the activation of ERK1/2 and inhibited the protein levels of E-cadherin and claudin-1. In addition, inhibition of ERK1/2 neutralized the effects of 5-FU on the protein levels of E-cadherin and claudin-1. These events are indications of prompt TGF-β-mediated EMT signaling via ERK1/2. EMT is marked by decreased E-cadherin expression which is, in turn, regulated by transcriptional regulators such as slug, snail1, and ZEB1.^36^ However, our present study indicates that these molecules are not involved in 5-FU-stimulated TGF-β signaling because their levels remained unaffected upon stimulation by 5-FU. Further research is needed to confirm these results and to clarify the roles of other EMT transcriptional regulators like TWIST1, TCF4, and TCF3^36^ in the context of how 5-FU stimulation affects EMT signaling. In addition to the data on signaling, we also observed that the morphology of Hep G2 colonies underwent an EMT-like change, and their invasion and migration accelerated, upon a 24-h treatment with the culture supernatant of 5-FU-treated cells. We detected secretion of TGF-β in the latter cells (data not shown). Further investigations are ongoing on factors that reduce the cytotoxic effects of the reagents and to obtain a more direct proof of 5-FU-induced EMT. These investigations include screening of cells to detect resistance to the cytotoxic activity of anti-tumor reagents, determination of the best dose and period of treatment with 5-FU, and whether to administer 5-FU in combination with IFNα-2b.

As we presently confirmed, IFNα inhibits the expression of TGF-β^37^. We provide the first evidence that IFNα-2b can antagonize the effects of 5-FU on TGF-β expression and TGF-β-mediated signaling. Therefore, it can be assumed that the higher efficacy of the combination therapy for advanced HCC happened by targeting both TGF-β and its downstream signals. Indeed, medical insurance does not cover the use of IFNα-2b in treatment of advanced HCC. Cisplatin^38^, sorafenib^39^ and more recently, the TGF-β receptor I kinase inhibitor, LY2157299 monohydrate^40^, have good efficacy against advanced HCC. The present data indicate that, except for IFNα-2b, co-treatment involving 5-FU and other compounds or inhibitors of TGF-β and its signaling will be efficacious. This is consistent with a prior study that described the therapeutic value of the combination of a TGF-β receptor kinase inhibitor with the 5-FU analog, S1, for the treatment of scirrhous gastric carcinoma with lymph node metastasis^41^.

Presently, TGF-β expression and secretion were unaffected by 5-FU treatment of normal hepatocytes, compared to Hep G2 and HuH-7 hepatoma cells. Moreover, the two hepatoma cell lines responded differently to treatment with 5-FU alone or in combination with IFNα-2b. The collective data indicate a cell-dependent function of 5-FU. Interestingly, the Hep G2 cell line was established from a HBV-positive male with a primary hepatoma^42, 43^, while HuH-7 cells possess HCV replicons capable of auto-replication^44^. Thus, our in vitro data might explain the variation in the clinical efficacy of the combination therapy. In other words, the efficacy of the therapy may differ between patients with HCV or HBV background. It has been reported that patients with HCC and a HCV-positive background had higher survival rates and longer survival periods than patients who were HCV-negative or produced higher levels of TGF-β receptor 2^45^. Furthermore, in contrast to Hep G2 cells, 5-FU increased the levels of p-SAMD2 and p15INK4b, and maintained or even increased the level of E-cadherin in HuH-7 cells. These observations indicate that apoptosis signaling increased in 5-FU-treated HuH-7 cells in contrast with Hep G2 cells. In cases with an HCV background that were analyzed, although the serum levels of TGF-β decreased after the combination therapy in all the patients, 41.2% of the cases responded poorly to the therapy with a continued worsening of their condition, and there were no significant differences in TGF-β levels between patients who worsened and improved. These data suggest that excessive inhibition of TGF-β and its signal may weaken the apoptotic function of 5-FU in patients with an HCV background, which could lead to poor or no response to the combination therapy.

In Japan and China, the majority of HCC patients have an HCV and HBV background, respectively. Proper treatment of these patients will undoubtedly determine the efficacy of HCC treatment, especially for those in advanced stages. The present data clarify the various effects of 5-FU and IFNα-2b applied alone and in combination, on the regulation of TGF-β levels and its signals in two hepatoma cells. The knowledge gained will aid in determining the mechanism of the combination chemotherapy, which will in turn drive the formulation of better therapies for advanced HCC.

## Materials and methods

### Subjects

Fifty patients with advanced HCC (stage IV-A/B) were treated using a subcutaneous administration of polyethylene glycol (PEG)-IFNα-2b (50–100 μg on day 1 of each week for 4 weeks) and intra-arterial infusion of 5-FU (250 mg/day for 5 h on days 1–5 of each week for 4 weeks). Blood was withdrawn before and after the therapy and centrifuged at 1500 × *g* for 10 min. The resulting serum was stored in aliquots at −80 °C. The largest tumor diameter was also determined in the patients. Approval for the study was obtained from the institutional review board (H19-87) of Iwate Medical University, Morioka, Japan, and informed consent was obtained from the patients’ relatives.

### Cells

Human HuH-7 and Hep G2 hepatocarcinoma cells were purchased from the American Type Culture Collection (ATCC, Manassas, VA, USA). The cells were cultured in Dulbecco’s modified Eagle’s medium (DMEM; Thermo Fisher Scientific, Rockford, IL, USA) containing 10% fetal bovine serum, (FBS; Thermo Fisher Scientific) and 100 U/mL penicillin–streptomycin (Sigma-Aldrich, St. Louis, MO, USA). Mouse AML-12 hepatocytes were kindly supplied by Professor Itaru Kojima, Gunma University, Japan. The cells were cultured in DMEM and Ham’s F-12 nutrient mixture. (1:1, Thermo Fisher Scientific) containing 10% FBS until 80% confluency was reached. After starvation, the cells were treated with 5-FU (Kyowa Kirin, Tokyo, Japan), IFNα-2b (Funakoshi Co., Ltd., Tokyo, Japan), or the combination of 5-FU and IFNα-2b in the absence or presence of an inhibitor of TGF-β. Some experiments also included SB-431542, which is the specific inhibitor of receptor kinase (Sigma-Aldrich), and U0126, which inhibits ERK kinases (MEK; Promega Corporation, WI, USA).

### Analysis of the levels of TGF-β1

TGFβ-1 levels were assessed in patients, before and after administering therapy, and in the cell culture supernatant of hepatoma cells using the Quantikine Human TGF-β1 Immunoassay Kit (R&D Systems Inc., Minneapolis, MN, USA). TGFβ-1 levels in the cell culture supernatant of AML-12 cells were assessed using the Quantikine Mouse/Rat/Porcine/Canine TGF-β1 Immunoassay Kit (R&D Systems, Inc., Minneapolis, MN, USA).

### Western blot analysis

Total protein was isolated from hepatocytes using a total protein extraction kit from the BioChain Institute (Newark, CA, USA) according to the manufacturer’s instructions. Ten micrograms of protein from each sample was separated using 10% sodium dodecyl sulfate polyacrylamide gel electrophoresis (SDS-PAGE) and electro-transferred to a polyvinylidene difluoride membrane. Immunoblotting was performed using antibodies from the Epithelial–mesenchymal Transition (EMT) Antibody Sampler Kit and specific antibodies against TGF-β, phospho (p)-SMAD2, SMAD2, SMAD4, p15INK4b, p-ERK1/2 (Cell Signaling Technology Japan, K.K., Tokyo, Japan). The antibody against β-actin was purchased from Santa Cruz Biotechnology, Santa Cruz, CA, USA. All antibodies showed reactivity to mouse and human cells. The immuno-reactive bands were visualized with an enhanced chemiluminescence reagent (GE Healthcare, Little Chalfont, Buckinghamshire, UK) and quantified with Image J software.

### Cell viability assay

Hep G2 cells were treated for 48 h under predetermined conditions. The number of viable cells was evaluated by adding SF cell counting reagent (Nacalai Tesque, Tokyo, Japan) directly to the cells. Absorbance was measured at 450 nm (A_450_) using an Immuno-Mini NJ-2300 microplate reader (Inter Med, Tokyo, Japan).

### Evaluation of apoptosis of Hep G2 cells

Hep G2 cells were treated for 48 h under predetermined conditions. Apoptotic cells were stained with 4′,6-diamidino-2-phenylindole dihydrochloride (DAPI; Dojindo, Japan). The cells were scored under an inverted fluorescence microscope (ECLIPSE TE300, Nikon, Japan) at 20 × magnification, and were photographed using a digital camera (DXC-S500/OL, Olympus, Tokyo, Japan). Apoptotic cells and non-apoptotic cells present in three randomly chosen microscopic fields were counted, and the percentage of apoptotic cells was calculated.

### Statistical analysis

Statistical analysis was carried out using *t*-test (Fig. 1b; Supplemental Data) and one-way ANOVA-POST HOC (Tukey’s HSD) analysis (Fig. 3a, c, SPSS statistics 17.0 software package, SPSS Japan Inc.). A *p*-value < 0.05 was considered significant. Data are presented as the mean ± standard deviation (SD).

## Electronic supplementary material

Supplement Data

Supplement Data Caption
