# Supplementary figures and images for: Regulation of transforming growth factor is involved in the efficacy of combined 5-fluorouracil and interferon alpha-2b therapy of advanced hepatocellular carcinoma
Source: Cell Death Discov. 2018 Mar 12;4:42. doi: 10.1038/s41420-018-0040-y (PMC5849890; doi:10.1038/s41420-018-0040-y)

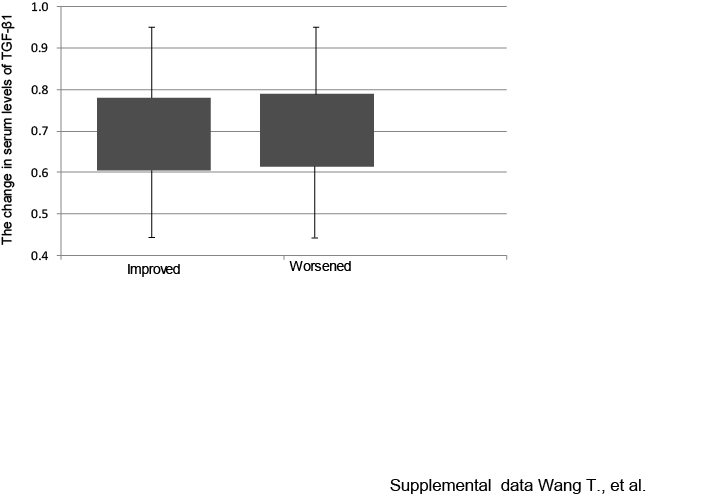

Supplement: Supplementary file 1 — Supplement Data [file 41420_2018_40_MOESM1_ESM.png]
