# Supplementary material for: Regulation of transforming growth factor is involved in the efficacy of combined 5-fluorouracil and interferon alpha-2b therapy of advanced hepatocellular carcinoma
Source: Cell Death Discov. 2018 Mar 12;4:42. doi: 10.1038/s41420-018-0040-y (PMC5849890; doi:10.1038/s41420-018-0040-y)
Supplement: Supplementary file 2 — Supplement Data Caption [file 41420_2018_40_MOESM2_ESM.docx]

Supplemental Data The change in serum levels of TGF-β1 with the combination therapy in the patients with an HCV background whose condition worsened (n=7) or improved (n=10). The change in serum levels of TGF-β1 was determined by calculating the ratio of serum level of TGF-β after the therapy with that before the therapy. The efficacy of the therapy was evaluated based on the results of CT scans one month after the therapy. The statistical analysis was carried out using t-test for unpaired comparisons. A *p* value <0.05 was considered to be significant. Results are expressed as the means ± SD. Seventeen patients were analyzed.
